# Ransomware Attacks and Data Breaches in US Health Care Systems

**DOI:** 10.1001/jamanetworkopen.2025.10180

**Published:** 2025-05-14

**Authors:** John Xuefeng Jiang, Joseph S. Ross, Ge Bai

**Affiliations:** 1 Accounting & Information Systems, Eli Broad College of Business, Michigan State University, East Lansing; 2 Yale School of Medicine, Yale School of Public Health, Yale University, New Haven, Connecticut; 3 Johns Hopkins Carey Business School, Johns Hopkins Bloomberg School of Public Health, Washington, DC

## Abstract

This cross-sectional study analyzes ransomware attacks across all HIPAA-covered entities from 2010 to 2024 and examines their contribution to protected health information data breaches.

## Introduction

Ransomware attacks, which restrict data access and encrypt information unless ransom payments are made, increasingly threaten health care operations.^1^ In February 2024, a ransomware attack on Change Healthcare compromised the protected health information (PHI) of 100 million individuals, disrupted care delivery nationwide, and incurred $2.4 billion in response costs.^2^

Although hacking or information technology (IT) incidents became the leading cause of health care data breaches in 2017, the proportion involving ransomware remains unclear.^3^ Prior research identified 376 ransomware attacks on health care delivery organizations from 2016 to 2021,^4^ but health plans and clearinghouses have also been victims. This study analyzes ransomware attacks across all Health Insurance Portability and Accountability Act (HIPAA)–covered entities from 2010 to 2024 and examines their contribution to PHI data breaches.

## Methods

This cross-sectional study used nonidentifiable public data and did not constitute human participant research; therefore, institutional review board approval was not required in accordance with the Common Rule. This study follows STROBE reporting guideline. We analyzed breaches affecting 500 or more patient records reported to the US Department of Health and Human Services (HHS) Office for Civil Rights (OCR) from October 2009 through October 2024. Data were obtained from the publicly available Breach Portal (eAppendix in Supplement 1). After removing duplicates and incomplete entries, 6468 unique breaches remained. Breaches were classified by reporting year (not occurrence year), acknowledging HIPAA’s 60-day reporting window. The OCR categorized breaches into hacking or IT incidents, theft, unauthorized access/disclosure, and improper disposal or loss, as well as breaches of unidentified or unknown cause.^5^

Breach details came from OCR records for fully investigated cases and web searches for ongoing cases (primarily 2023-2024).^6^ According to OCR’s classification, cyber intrusions are categorized as a hacking or IT incident. We identified ransomware attacks within this category by analyzing event descriptions for specific indicators, including ransom demands, cryptocurrency payments, system encryption, or known ransomware groups (eg, LockBit, BlackCat). Details are provided in the eAppendix in Supplement 1. The frequency and the number of affected records across 5 breach categories—ransomware hacking or IT incidents, nonransomware hacking or IT incidents, theft, unauthorized access or disclosure, and improper disposal or other breaches—were analyzed. The analyses were conducted using SAS, version 9.4.

## Results

The total number of PHI data breaches increased from 216 in 2010 to 566 in 2024, with hacking or IT incidents increasing from 4% (8 of 216) to 81% (457 of 566) of all breaches (*P* < .001) (Table). Ransomware attacks increased from 0 cases in 2010 to 31% (222 of 715) of breaches in 2021, before decreasing to 11% (61 of 566) in 2024. Concurrently, breaches due to theft, unauthorized access, and improper disposal or loss decreased (Figure, A).

**Table.** Trends in Hacking or IT Incidents and Ransomware Data Breaches in Health Care, 2010-2024^a^

Year	Breaches			Records affected^b^		
	Total No.^c^	Hacking or IT, No. (%)^d^	Ransomware, No. (%)^e^	Total No.	Hacking or IT, No. (%)	Ransomware, No. (%)
2010	216	8 (4)	0	6066	92 (2)	0
2011	200	17 (9)	1 (1)	13 162	298 (2)	3 (0.02)
2012	218	17 (8)	2 (1)	2855	908 (32)	35 (1)
2013	276	29 (11)	2 (1)	7019	298 (4)	11 (0.2)
2014	314	39 (12)	1 (0.3)	19 074	7991 (42)	4 (0.02)
2015	269	55 (20)	3 (1)	112 466	110 971 (99)	16 (0.01)
2016	328	114 (35)	30 (9)	16 711	13 482 (81)	324 (2)
2017	358	149 (42)	58 (16)	5315	3697 (70)	1887 (36)
2018	369	165 (45)	37 (10)	15 236	11 267 (74)	2800 (18)
2019	511	314 (61)	72 (14)	44 970	40 992 (91)	4739 (11)
2020	663	457 (69)	203 (31)	35 310	32 628 (92)	18 176 (51)
2021	715	547 (77)	222 (31)	60 193	58 045 (96)	26 754 (44)
2022	720	570 (79)	204 (28)	57 665	49 807 (86)	29 246 (51)
2023	745	602 (81)	165 (22)	166 504	158 009 (95)	84 491 (51)
2024	566	457 (81)	61 (11)	170 001	154 616 (91)	116 946 (69)
Total, No.	6468	3540	1090	732 546	643 100	285 431

^a^ Data for 2024 are incomplete because the sample period ended on October 31, 2024, and should be interpreted with caution because they do not represent complete annual trends.

^b^ Represents the number of records affected by all data breaches, hacking, or information technology (IT) incidents and ransomware attacks, respectively.

^c^ The total number of data breaches reported to the Office for Civil Rights that affected 500 or more individuals’ electronic protected health information as required under the Health Insurance Portability and Accountability Act, as amended by the Health Information Technology for Economic and Clinical Health Act.

^d^ The number of breaches attributed to hacking or IT incidents.

^e^ The number of ransomware attacks, representing a subset of hacking or IT incidents.

**Figure.**
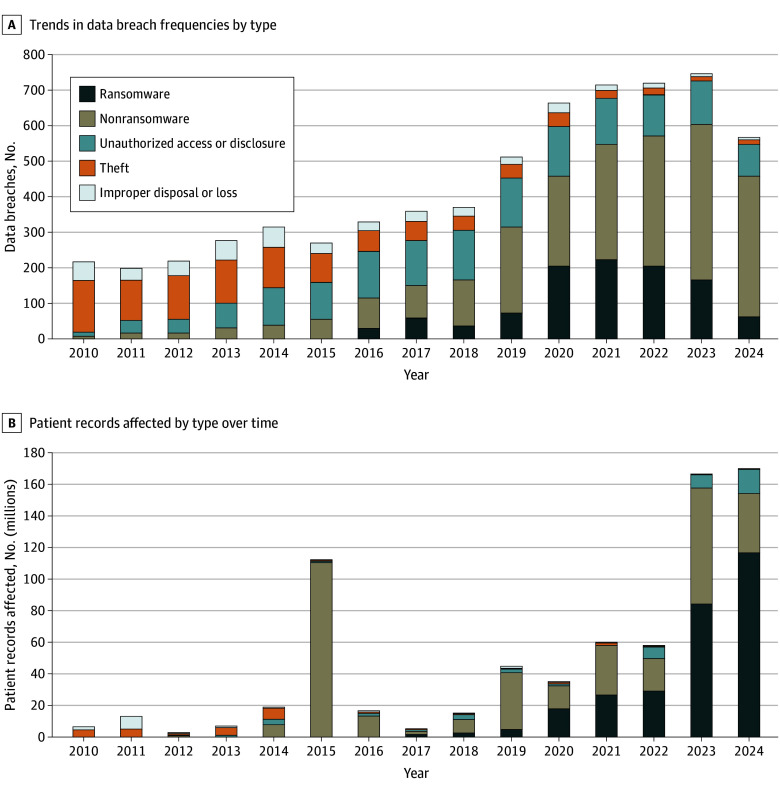
Trends in Health Care Data Breaches and Affected Patient Records, by Breach Type A, Improper disposal and other breaches include those of unidentified or unknown causes. The breaches in 2024 are incomplete because our sample period ended on October 31, 2024. B, Improper disposal or loss breaches include those of unidentified or unknown causes. The spike in 2015 was driven by a cyberattack on Anthem, which exposed the electronic protected health information of 79 million patients records.

The number of patient records affected by PHI data breaches increased from 6 million in 2010 to 170 million in 2024, with hacking or IT incidents increased from 2% (92 358 of 6 million) to 91% (155 million of 170 million). Of the 732 million records affected from 2010 to 2024, hacking or IT incidents and ransomware accounted for 88% (643 million) and 39% (285 million), respectively. Since 2020, ransomware has affected more than half of all patients annually, reaching 69% in 2024 (Figure, B).

## Discussion

Health care PHI data breaches surged from 2010 to 2024, driven by hacking or IT incidents, particularly ransomware attacks. Consistent with HHS breach assessments and prior literature, we measure breach impact by the number of patient records affected. However, this study is limited in that this metric may not fully reflect ransomware’s operational disruptions. Additionally, our findings likely underestimate the frequency of data breaches due to underreporting, reluctance to disclose ransom payments, and the OCR’s exclusion of breaches affecting fewer than 500 records.

Hospitals, clinics, health plans, and other HIPAA-covered entities are particularly vulnerable to ransomware attacks due to limited cybersecurity resources and the urgency of system recovery for patient care. Mitigation strategies should include mandatory ransomware fields in OCR reporting to improve surveillance clarity, revising severity classifications to account for operational impact, and monitoring cryptocurrency to disrupt ransom payments.

## Notes

Supplement 1. **eAppendix.** Ransomware Classification Details

Supplement 2.
Data Sharing Statement
