# Supplementary material for: Ransomware Attacks and Data Breaches in US Health Care Systems
Source: JAMA Netw Open. 2025 May 14;8(5):e2510180. doi: 10.1001/jamanetworkopen.2025.10180 (PMC12079295; doi:10.1001/jamanetworkopen.2025.10180)
Supplement: Supplement 1. — eAppendix. Ransomware Classification Details [file jamanetwopen-e2510180-s001.pdf]

Supplemental Online Content

Jian JX, Ross JS, Bai G, et al. Ransomware attacks and data breaches in US health care systems. *JAMA Netw Open*. 2025;8(5):e2510180. doi:10.1001/jamanetworkopen.2025.10180

eAppendix. Ransomware Classification Details

This supplemental material has been provided by the authors to give readers additional information about their work.

© 2025 Jian JX et al. *JAMA Network Open*

eAppendix. Ransomware Classification Details

We developed a systematic, rule-based algorithm in Python to identify ransomware attacks among healthcare data breaches. Our natural language processing approach applies regular expression pattern matching to categorize incidents based on specific textual indicators found in breach descriptions.

Data Sources

We analyzed breach descriptions from two primary sources:

- Official case descriptions from the HHS Office for Civil Rights (OCR) breach portal. This publicly available database can be accessed at: https://ocrportal.hhs.gov/ocr/breach/breach_report.jsf.
- Media reports obtained through google searches for incidents where official descriptions provided insufficient detail

Primary Ransomware Indicators

Our algorithm employs a two-tier classification system with decreasing specificity:

Direct Ransomware Identification

- Primary pattern: Explicit mention of "ransomware" or "ransom" terms
- Negative filter: Exclusion of cases with explicit negation patterns (e.g., "not ransomware", "no ransom")

Secondary Ransomware Indicators

- Cryptocurrency references: "bitcoin", "crypto"
- Encryption evidence: "locked", "encrypt"
- Extortion language: Patterns like "demand.*payment", "threaten.*pay", "extort" (with negative lookahead filters to exclude regulatory actions)
- Known threat actors: Specific ransomware group identifiers including "lockbit", "blackcat", "alphv", "conti", "ryuk", "revil", "darkside", "hive", "clop", "ransomhub"

Non-Ransomware Classification

When ransomware indicators are absent, we classify incidents based on other cybersecurity patterns:

- Attack vectors: "phish", "spoofed", "email.*compromise", "zero-day", "vulnerability", "exploit", "cve-\d+"
- Data compromise patterns: "stole.*data", "exfiltrated", "exposed.*data", "accessed.*database"
- System impact terminology: "intrusion", "hacked", "compromised", "malware", "virus", "trojan"
- Service effects: "disrupted.*operations", "systems.*down", "service.*interrupted"

Classification Precision Measures

To minimize false positives and ensure classification accuracy:

© 2025 Jian JX et al. *JAMA Network Open*

- Regular expressions include negative lookaheads (e.g., "(?!.*ransom)") to prevent misclassification
- Cases with ambiguous or insufficient detail are explicitly classified as "Uncertain" and not counted as ransomware incidents
- Regulatory terminology like "fine", "settlement", "department", "agency" is filtered from extortion pattern matching
- Incidents tagged as "Uncertain" are treated as non-ransomware to ensure conservative estimation

Our approach likely underestimates the true prevalence of ransomware attacks in healthcare, making our findings a lower-bound estimate of the ransomware impact.

© 2025 Jian JX et al. *JAMA Network Open*
