# Supplementary material for: Ransomware Attacks and Data Breaches in US Health Care Systems
Source: JAMA Netw Open. 2025 May 14;8(5):e2510180. doi: 10.1001/jamanetworkopen.2025.10180 (PMC12079295; doi:10.1001/jamanetworkopen.2025.10180)
Supplement: Supplement 2. — Data Sharing Statement [file jamanetwopen-e2510180-s002.pdf]

Data Sharing Statement

Jiang. Ransomware Attacks and Data Breaches in US Health Care Systems. *JAMA Netw Open*. Published May 14, 2025. doi:10.1001/jamanetworkopen.2025.10180

Data
Data available: No

Additional Information
Explanation for why data not available: Data for this analysis are publicly available from the U.S. Department of Health and Human Services (HHS) Office for Civil Rights (OCR) "Breach Portal: Notice to the Secretary of HHS Breach of Unsecured Protected Health Information" (https://ocrportal.hhs.gov/ocr/breach/breach_report.jsf). Our ransomware classification methodology is included in the supplement.The numerical statistics underlying the figures not displayed in the manuscript will be made available upon request to the corresponding author.
